# Silicone Oil Reinjection without Macular Buckling for Treatment of Recurrent Myopic Macular Hole Retinal Detachment after Silicone Oil Removal

**DOI:** 10.1155/2014/434272

**Published:** 2014-02-11

**Authors:** Hammouda Hamdy Ghoraba, Sameh Mohamed Elgouhary, Hosam Osman Mansour

**Affiliations:** ^1^Tanta University, Tanta 31111, Egypt; ^2^Maghrabi Eye Hospital-Tanta Branch, Tanta 31517, Egypt; ^3^Department of Ophthalmology, Menoufia University, Shebin Elkom, Menoufia 32511, Egypt; ^4^Alazhar University-Damietta Branch, Damietta 34519, Egypt

## Abstract

*Purpose*. To evaluate the efficacy of silicone oil (S.O) reinjection without macular buckling for treatment of recurrent myopic macular hole retinal detachment (MHRD) after silicone oil removal. *Methods*. A retrospective consecutive interventional study from medical reports on cases of myopic MHRD. Fifty-three eyes of 51 patients underwent silicone oil removal after successful repair of MHRD were reviewed. The main outcomes were the retinal status after silicone oil removal and management of recurrent cases. *Results*. The rate of recurrent RD (Re RD) after silicone oil removal was 11.3% (6 out of 53 eyes). One case refused any other interference. In the remaining 5 eyes, 4 eyes (80%) could be reattached by S.O re-injection and one eye (20%) developed Re RD after S.O re-injection. Range of followup after management of recurrence was 5–53 months (mean 18.7 months). *Conclusions*. This case series concluded that the risk factors for recurrent RD after silicone oil removal from cases of myopic MHRD were high myopia, open flat MH, and large posterior staphyloma. Revision of vitrectomy and S.O re-injection can reattach most of recurrent cases.

## 1. Introduction

Macular hole retinal detachment (MHRD) is defined as retinal detachment without associated peripheral breaks [1]. Retinal detachment secondary to macular hole during the degenerative changes of highly myopic eyes is one of the most important causes of blindness or loss of central vision. Highly myopic eyes are often accompanied by pathologic fundus changes, such as posterior pole chorioretinal atrophy and posterior staphyloma [2].

Several procedures were introduced for the repair of MHRD, including primary gas tamponade, pars plana vitrectomy (PPV) with gas tamponade, silicone oil (S.O) tamponade, and internal limiting membrane (ILM) dissection [3, 4].

Since recurrent retinal detachments after silicone oil removal in myopic eyes are not scarce, it is difficult for surgeons to evaluate the appropriate time to remove silicone oil. There is no available information regarding the possibility of successful silicone oil removal associated with the postoperative macular configuration [5].

The recent literatures [6–8] discuss macular buckling as a treatment option for myopic MHRD particularly if recurrent. The rarity of details about the management of recurrent myopic MHRD after silicone oil removal encouraged us to review our cases.

## 2. Patients and Methods

This was a retrospective review of consecutive 53 eyes that underwent silicone oil removal after successful repair of myopic macular hole retinal detachment. The following data were extracted from medical records of the included patients: age, gender, best corrected visual acuity, slit lamp examination of the anterior segment, intraocular pressure measurement, fundus examination using +90D lens and indirect ophthalmoscope, coloured fundus photography, B-scan U/S, and OCT. All surgeries were performed by a single surgeon (HG) at a single center.

Exclusion criteria were (1) previous vitreoretinal surgery, (2) MH associated with tractional RD, (3) traumatic MH, (4) myopic foveoschisis, (5) and less than 3 months of followup after silicone oil removal.

After discussing the nature of surgery with the patients including the potential risks and complications, all patients signed a written informed consent document before surgery.

### 2.1. Primary Surgery

The primary surgery consisted of 3-port pars plana vitrectomy (PPV) using conventional 20 gauge system or transconjunctival system (20, 23, and 25 gauge or a combination of them). Vitreous surgery was the same in all eyes and consisted of core vitrectomy; triamcinolone acetonide (TA) assisted in removal of posterior hyaloid. TA was used multiple times to ensure complete removal of posterior hyaloid. Trial of ILM peeling from macular arcade to the other was attempted in all eyes with the aid of TA and using Eckardt forceps. ILM peeling was identified by whitening of retinal surface and petechial hemorrhages. We did not use dye to assist ILM peeling for fear of its escape into the subretinal space through the MH with potential toxicity. Drainage of subretinal fluid was done from the MH during fluid-air exchange. This was done multiple times (because of fluid recollection) until complete dryness. No laser was applied to the macular hole rim in any of the included eyes. Silicone oil 5000 or 2000 centistokes tamponade was used at the end of surgery. Patients were instructed to maintain a prone position for 8 hours/day for at least 1 week postoperatively.

Removal of silicone oil from successfully reattached retina was done using 2 conventional 20 gauge openings and 20 or 23 gauge transconjunctival systems. Phacoemulsification and intraocular lens implantation was performed for phakic patients. Silicone oil was aspirated mechanically or left to be escaped from the 20- or 23-gauge cannula. We thoroughly reexamined the retina after complete silicone oil removal. This was followed by multiple air-fluid exchange to decrease the amount of residual emulsified silicone oil. Sclerotomies were self-sealed or sutured using vicryl 7/0 sutures (when needed).

### 2.2. Management of Recurrence after Silicone Oil Removal

Once identified, recurrent cases were advised to have a revision surgery. In this resurgery, triamcinolone acetonide (TA) was injected and then washed out. Examination of the macula using Plano concave lens with high magnification was done. We searched for the possible causes of recurrence and treated it if identified. If epimacular membrane was identified, it was peeled with the aid of TA using Eckardt forceps. If no cause other than MH was identified, air-fluid exchange and drainage of subretinal fluid through the MH were done. Silicone oil 5000 centistokes was injected at the end of surgery.

The statistical analysis was performed using a commercially available statistical software package (SPSS for windows, version 12.0).

## 3. Results

This was a retrospective review on 53 eyes of 51 patients who underwent silicone oil removal after successful repair of myopic MHRD.

### 3.1. Patients' Data

There were 29 (56.9%) females and 22 (43.1%) males. The range of age was 27–74 years (mean, 54.9 years). Range of myopia was from 4.5 to 25 diopters. The mean interval between the first surgery and silicone oil removal was 16.4 months (range, 4–74 months). Range of followup after silicone oil removal was 5–74 months (mean, 23 months). Range of followup after management of recurrence was 5–53 months (mean, 18.7 months).

### 3.2. Recurrent RD after Silicone Oil Removal

Recurrent retinal detachment after silicone oil removal occurred in 6 eyes out of total 53 eyes (11.3%). Recurrences were observed at variable periods after silicone oil removal. Recurrence occurred after 2 weeks in 2 eyes (33.4%), 1 month in one eye (16.6%), 2 months in 2 eyes (33.4%), and 14 months in one eye (16.6%). All the recurrent cases had open MH, high myopia more than 12 diopters, and posterior staphyloma.

From the six cases that had recurrent RD after silicone oil removal, one patient refused resurgery and the other 5 patients accepted resurgery. Of these 5 cases, the retina of 4 eyes remained flat under silicone oil (80%) and one eye developed central Re RD under silicone oil (20%) and refused any other interference.

Silicone oil was retained in the successfully attached cases after silicone oil reinjection because of patient refusal to remove silicone oil in 3 cases, and the fourth case was single eyed patient with no silicone oil induced IOP rise.

### 3.3. OCT Findings

OCT (*HD cirrus OCT*) evaluation of the macula was done one week after silicone oil removal.

**Figure 1:**
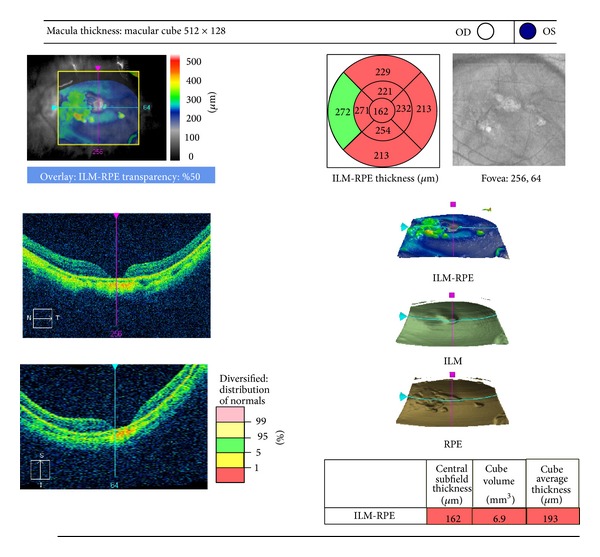
OCT picture of flat open macular hole of a male patient of 50 years old with recurrent RD after silicone oil removal.

OCT findings were as follows: flat open macular hole in 32 eyes (60.4%), closed MH in 20 eyes (37.8%), and macular scar in one eye (1.8%) (Figure 1).

## 4. Discussion

Nowadays, there is a debate about treatment of recurrent myopic macular hole RD after silicone oil removal. The available treatment options are silicone oil reinjection, macular buckling, or combined macular buckling, and silicone oil reinjection.

In this case series, we tried to evaluate the efficacy of silicone oil reinjection without macular buckling for treatment of recurrent myopic macular hole retinal detachment after silicone oil removal.

Silicone oil reinjection is a simple technique and can be done by all vitreoretinal surgeons but it has the disadvantages of the possibility of recurrent RD after silicone oil removal (especially in the presence of high myopia, posterior staphyloma, and open and large macular hole) and complications may occur as emulsification of silicone oil and increase of intraocular pressure. Macular buckling (as recommended by some authors [6–8]) may be considered in these cases; however it cannot guarantee a 100% success rate in addition to being a difficult technique that needs a prolonged period of learning curve. Combined macular buckling and silicone oil reinjection may have a better success rate with more safe silicone oil removal.

In this case series, the rate of recurrent RD after silicone oil removal was 11.3% (6/53). We found that the risk factors for Re RD were open flat holes, high myopia, and posterior staphyloma. Five of the recurrent cases had a resurgery and silicone oil reinjection. Four of them could be reattached (Table 1).

We think that multiple air-fluid exchange during treatment of recurrence may lead to stretching of the retina and regaining of some retinal attachment. Also we think that silicone oil prevents fluid currents in the vitreous cavity; this may prevent recurrent RD under silicone oil.

Nadal et al., 2012 [9], reported a recurrence rate of 14.9% (4/27) under silicone oil. These 4 cases were treated by macular indentation with subsequent silicone oil removal. They did not give any comment about the retinal state of these 4 cases after silicone oil removal or the technique of macular indentation they used. Qian and Jiang, 2010 [5], reported recurrence rate of 19.5% (8/41) after silicone oil removal of successful cases (41/51) without details about how they managed the recurrence after silicone oil removal.

In our work, the recurrence in relation to the MH status (proved by OCT) revealed no recurrent retinal detachment after silicone oil removal if the MH was closed. Recurrence occurred in 6 out of 32 eyes with flat open macular hole (18.7%). Qian and Jiang, 2010 [5], reported recurrent retinal detachment in 8 out of 21 eyes with flat open macular hole (38%).

In this study, recurrent retinal detachment after silicone oil removal in cases of MHRD occurred in highly myopic eyes with open flat MH and posterior staphyloma. These factors were also reported by Nadal et al., 2012 [9]. Qian and Jiang, 2010 [5], reported that open MH was the main risk of recurrence.

To our knowledge, there was no data in the literature discussing the effect of the size of MH, state of posterior pole atrophy, or the depth of posterior staphyloma as possible factors affecting the success of management of macular hole RD and this may be a good point for further research.

Limitations of this study were the retrospective nature of data collection and that silicone oil was retained in successfully reattached cases. Further study to compare silicone oil reinjection and macular buckling in the management of recurrent RD after silicone oil removal in cases of MHRD could be of great value.

Finally, we recommend that for treatment of recurrent myopic MHRD after silicone oil removal, macular buckling may be considered (to relieve the tangential traction to the edges of MH caused by posterior staphyloma) either alone or combined with silicone oil reinjection (to prevent fluid currents in the vitreous cavity) with further silicone oil removal. Laser to the edge of MH combined with gas injection may be considered during silicone oil removal especially in the presence of poor visual acuity or posterior pool atrophy.

## Figures and Tables

**Table 1:** Summary of recurrent cases.

Number	Age	Gender	Myopia in diopters	Posterior staphyloma	1ry surgery	Interval between 1ry surgery and SOR	Time of recurrence	OCT after SOR	Management of recurrence	Current retinal state after resurgery	Followup	Final V/A
1	67	Female	12.0	Present	SB + PPLV + S.O	18 months	2 months	Flat open MH	S.O injection	Flat	53 months	20/200
2	74	Female	14.0	Present	SB + PPV + S.O	17 months	1 month	Flat open MH	S.O injection	Re RD under S.O	13 months	CFs at 50 cm
3	49	Female	20.0	Present	PPV + S.O	13 months	2 weeks	Flat open MH	S.O injection	Flat	13 months	CFs at 1 meter
4	50	Male	14.0	Present	PPV + ILM + S.O	12 months	2 months	Flat open MH + EMM	MP + S.O injection	Flat	8 months	CFs at 1 meter
5	30	Female	21.0	Present	SB + PPV + S.O	10 months	14 months	Flat open MH	S.O injection	Flat	10 months	20/400
6	60	Male	17.0	Present	Phaco + SB + PPV + S.O	12 months	2 weeks	Flat open MH	Refuse resurgery	Re RD and refused S.O reinjection	18 months	CFs at 50 cm

SOR: silicone oil removal. OCT: optical coherence tomography. MH: macular hole. EMM: epimacular membrane. SO: silicone oil. MP: membrane peeling. Re RD: recurrent retinal detachment. SB: encircling 360 degree scleral buckling. PPV: pars plana vitrectomy. PPLV: pars plana lensectomy and vitrectomy. Phaco: phacoemulsification. V/A: visual acuity. CFs: counting fingers.
